# Effectiveness of BCG vaccination to aged mice

**DOI:** 10.1186/1742-4933-7-12

**Published:** 2010-09-01

**Authors:** Tsukasa Ito, Takemasa Takii, Mitsuo Maruyama, Daisuke Hayashi, Takeshi Wako, Azusa Asai, Yasuhiro Horita, Keiichi Taniguchi, Ikuya Yano, Saburo Yamamoto, Kikuo Onozaki

**Affiliations:** 1 Department of Molecular Health Sciences, Graduate School of Pharmaceutical Sciences, Nagoya City University, 3-1 Tanabe, Mizuho, Nagoya 467-8603, Japan; 2 Department of Mechanism of Aging, Institute for Longevity Sciences, National Center for Geriatrics and Gerontology, 35 Gengo, Morioka, Obu, Aichi 474-8522, Japan; 3 Japan BCG Central Laboratory, 3-1-5 Matsuyama, Kiyose, Tokyo 204-0022, Japan

## Abstract

**Background:**

The tuberculosis (TB) still increases in the number of new cases, which is estimated to approach 10 million in 2010. The number of aged people has been growing all over the world. Ageing is one of risk factors in tuberculosis because of decreased immune responses in aged people. *Mycobacterium bovis *Bacillus Calmette Guérin (BCG) is a sole vaccine currently used for TB, however, the efficacy of BCG in adults is still a matter of debate. Emerging the multidrug resistant *Mycobacterium tuberculosis *(MDR-TB) make us to see the importance of vaccination against TB in new light. In this study, we evaluated the efficacy of BCG vaccination in aged mice.

**Results:**

The Th1 responses, interferon-γ production and interleukin 2, in BCG inoculated aged mice (24-month-old) were comparable to those of young mice (4- to 6-week-old). The protection activity of BCG in aged mice against *Mycobacterium tuberculosis *H_37_Rv was also the same as young mice.

**Conclusion:**

These findings suggest that vaccination in aged generation is still effective for protection against tuberculosis.

## Introduction

The number of aged people has been increasing all over the world. World health organization reported that the increase rate of the number was 28% in the recent decade (2000 to 2009), and predicted that the number would reach 1.5 billion by 2050 [1]. The largest population of aged people (predicted to be 78% by the year 2050) resides in developing countries [1], where many infectious diseases, including tuberculosis (TB) [2], are endemic. Given the increased susceptibility of the elderly to infectious diseases, the rapid rise in the elderly population will become a significant threat to global health care.

The current TB vaccine is the live attenuated bacterium *Mycobacterium bovis *Bacillus Calmette Guérin (BCG). BCG is known to protect against tuberculous meningitis in babies children. However, it does not efficiently and consistently protect against pulmonary TB in adults. Over the years, many hypotheses have been put forward to explain the apparent variability in the protective efficacy of BCG, which varies from 0 to 80% [3]. Explanations for this inconsistency include differences in trial methodology, host population genetics, use of different BCG vaccine strains [4], and heterogeneous immunity to a variety of environmental mycobacteria that may interfere with the protection provided by BCG [5,6]. We have previously reported that 'early shared BCG strains' (ex. BCG-Russia, BCG-Moreau, BCG-Japan), which are chronologically early strains distributed form Pasteur Institute, conserve the characteristics of authentic BCG vaccine [7,8]. Immune response profiles following BCG vaccination comprise myriads of effecter mechanisms, multiple T-cell subsets, and many targeted antigens. BCG is capable of inducing Th1 responses [9], which are critical for protection against mycobacterial infection [10].

As an individual ages, significant immunological changes occur, which contribute to the enhanced morbidity and mortality associated with infectious diseases in the elderly. After puberty, thymic atrophy leads to a progressive decrease in the output of naïve T cells and decreased diversity in the T-cell repertoire [11]. CD8^+ ^T cells play an important role in innate response to pathogen such as *M. tuberculosis *in the aged mouse [12,13]. In this study, the efficacy of vaccination with BCG in aged mice was investigated.

## Results and Discussion

Specific-pathogen-free female C57BL/6 mice were supplied from the national center for geriatrics and gerontology (SLC, Japan). Young mice were 4- to 6-week of age, and old mice were 24-month of age. CD4/CD8 T cell ratio decreased by aging (Table 1), which is similar to previous reports [14,15]. Mice were vaccinated intraperitoneally with 10^6 ^colony forming unit (CFU) of *M. bovis *BCG Japan (Tokyo172) in 0.1 ml saline. The immunized mice were infected intravenously with 10^5 ^CFU *M. tuberculosis *strain H_37_Rv (ATCC 25618). To assess mycobacterial multiplication in spleen and lung, 0.1 ml of serial 10-fold dilutions of whole-organ 2-ml homogenates were plated onto Middlebrook 7H11 agar containing 10% OADC, and colonies were counted after 18-20 days post incubation at 37°C.

**Table 1:** T cell subsets in the peripheral blood of young and aged mice ^a^

	CD4	CD8	CD4/CD8
Young (4-6-week)	12.9 ± 6.55	17.3 ± 9.23	1.33 ± 0.66
Aged (24-month)	5.5 ± 1.83	6.2 ± 0.37	0.56 ± 0.28

^a ^PBMC were stained with FITC-labeled anti-CD4 and PE-labeled anti-CD8 mAbs (BD Biosciences) and analyzed by FACScan. Data represent mean ± of three mice.

**Figure 1:**
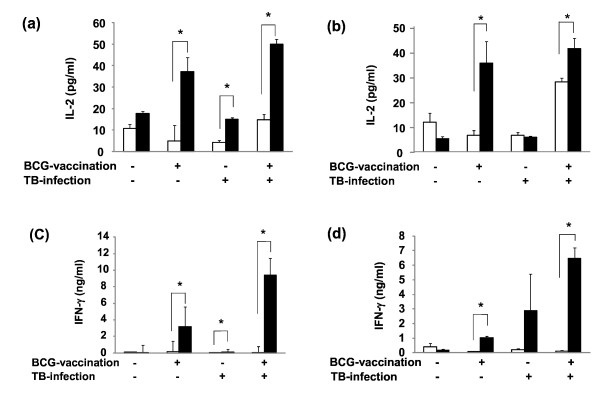
**BCG vaccination to young and aged mice augments Th1 cytokine production by PPD stimulated splenocytes**. Young (a, c) and aged (b, d) mice were immunized with 1 × 10^6 ^CFU of BCG or phosphate buffered saline (PBS). Eight weeks after immunization, the animals were intravenously challenged with 1 × 10^5 ^CFU of *M. tuberculosis *strain H_37_Rv. Four weeks after challenge, the levels of IL-2 (a, b) and IFN-γ (c, d) in the culture supernatants of splenocytes stimulated with PBS (open column) or PPD (solid column) were measured by ELISA (BD Biosciences). Data represent mean ± SD of three mice. *, *p *< 0.05, compared with PBS-stimulation (student's *t*-test).

Th1 type cytokines are known to play an important role in the cellular type immune response in tuberculosis [16,17]. We investigated whether the Th1 cytokines were induced by BCG vaccination. The productions of interferon (IFN)-γ and interleukin (IL)-2 from splenocytes stimulated with purified protein derivatives (PPD) were elevated in both BCG vaccinated young (4- to 6-month-old) and aged mice (24-month-old), but not in non vaccinated mice (Figure 1). These results indicate that BCG vaccination can induce Th1 responses in aged mice comparable to young mice.

In order to avoid the effects of cytokines induced by BCG vaccination on infection of *M. tuberculosis*, the levels of cytokines (IL-6, IL-10, IL-12p70, monocyte chemotactic protein (MCP)-1, IFN-γ, tumor necrosis factor (TNF)-α) in the serum from BCG vaccinated young mice were measured by Cytometric Beads Array (CBA) system (BD Biosciences, San Jose, CA). IL-10, IL-12 p70 and IFN-γ increased at 1 week post-inoculation, and returned to the basal level at 2 to 4 weeks. IL-6, TNF, MCP-1 peaked at two weeks after inoculation, and returned to the basal level at 4 weeks (data not shown). Therefore, the infection experiments were conducted six weeks after inoculation.

**Figure 2:**
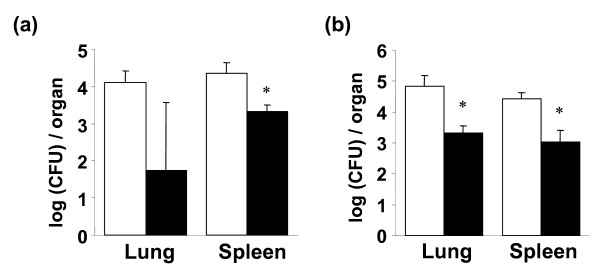
**BCG protects both young and aged mice against *M. tuberculosis *infection**. Young (a) and aged (b) mice were immunized with 1 × 10^6 ^CFU of BCG (solid column) or PBS (open column). Eight weeks after immunization, the mice were intravenously challenged with 1 × 10^5 ^CFU of *M. tuberculosis *strain H_37_Rv. Four weeks after challenge, the bacterial numbers in the lung and spleen were determined by colony assay. Data represent mean ± SD of three mice. *, *p *< 0.05, compared with PBS-inoculated control group (student's *t*-test).

To confirm the inoculation of BCG and infection of *M. tuberculosis*, the level of serum antibody against PPD was measured by ELISA. The level of the antibody elevated two weeks after BCG inoculation, peaked at 4 weeks and returned to the basal level at 6 weeks. After infection of *M. tuberculosis *the level was elevated again (data not shown). Body weights of young mice slightly increased according to their growth, while those of aged mice slightly decreased (data not shown). The changes in body weight of young and aged vaccinated-mice were the same as those of non-vaccinated mice. Vaccinated mice grew normally, and death was not observed in both young and aged mice. Therefore, vaccination of BCG would be safe to both young and aged mice. Eight weeks after inoculation of BCG, mice were infected with *M. tuberculosis *H_37_Rv intravenously. Four weeks after infection the mice were killed and colony assay for bacterial count in the organs (lung and liver) from young and aged mice was performed (Figure 2). The bacterial numbers in both young and aged mice were reduced. These data indicate that BCG vaccination could induce the protection against *M. tuberculosis *in aged mice to the same degree as young mice.

## Conclusion

In conclusion, this is a first report to evaluate the efficacy of BCG vaccination in aged (24-month-old) mice as compared to young (4- to 6-week-old) mice. BCG inoculation induced Th1 type immune responses in both young and aged mice (Figure 1). The protection activity was observed in aged mice, which was comparable to young mice (Figure 2). Our study suggests that vaccination in aged people could be effective to prevent infection against tuberculosis.

## Abbreviations

TB: tuberculosis; BCG: *Mycobacterium bovis *Bacillus Calmette Guérin; IL: interleukin; MCP: monocyte chemotactic protein; INF: interferon; TNF: tumor necrosis factor; CFU: colony forming unit; PPD: purified protein derivatives.

## Competing interests

The authors declare that they have no competing interests.

## Authors' contributions

TT, MM, and KO designed and planned the research. TI, TT, MM, TW, and AA performed the collection of serum and cytokine analysis. TI, DH, and YH performed infectious experiments and counting colonies of bacilli form infected organs. TI and KT performed FACS analysis. IY and SY supplied the BCG vaccine. All authors read and approved the final manuscript.
